# The associations among genetic features, late gadolinium enhancement and prognosis in hypertrophic cardiomyopathy

**DOI:** 10.3389/fcvm.2025.1597405

**Published:** 2025-07-07

**Authors:** Wenhua Su, Hao Wu, Chen Chen, Hongjiang Zhang, Qiuyue Yu, Liwen Liang, Qian Huo, Hongbo Lou, Bingjun Che, Yan Zhao, Juhua Dan, Hong Zhang

**Affiliations:** ^1^ Faculty of Life Science and Biotechnology, Kunming University of Science and Technology, Kunming, China; ^2^ Department of Cardiology, The First People's Hospital of Yunnan Province, Kunming, China; ^3^ Department of Cardiology, The People's Hospital of Kaizhou District, Chongqing, China; ^4^ Department of Magnetic Resonance, The First People’s Hospital of Yunnan Province, Kunming, China; ^5^ Department of Geriatrics, The First People’s Hospital of Yunnan Province, Kunming, China

**Keywords:** hypertrophic cardiomyopathy, late gadolinium enhancement, genotype, phenotype, major adverse cardiac and cerebrovascular events

## Abstract

**Aims:**

To assess the combined prognostic value of genotype and late gadolinium enhancement (LGE) in hypertrophic cardiomyopathy (HCM) patients, including those with preserved left ventricular ejection fraction (LVEF).

**Methods:**

In 135 HCM patients (age 52.43 ± 11.35 years, 79.26% male), whole-exome sequencing, echocardiography, and cardiac magnetic resonance (CMR) were performed. Major adverse cardiovascular and cerebrovascular events (MACCEs, e.g., cardiac death, progressive heart failure, sustained ventricular tachycardia/ventricular fibrillation, ICDs implantation, stroke, syncope, and atrial fibrillation) were analyzed over a median 15-month follow-up (IQR 9–36 months).

**Results:**

Pathogenic/likely pathogenic variants (G+) were identified in 50 (37%) patients, and LGE (L+) in 54 (40%). L+ patients exhibited worse clinical profiles: higher NYHA III–IV class (37% vs. 11%, *P* < 0.001), increased heart failure hospitalization (26% vs. 7%, *P* = 0.003), larger LV end-diastolic volume (median: 135, IQR: 125.25–213.00 vs. median: 126, IQR: 106.00–155.50, *P* = 0.004), lower LVEF (median: 55%, IQR: 39.75%–62% vs. median: 58%, IQR: 48%–65.5%, *P* = 0.012), and higher G+ prevalence (52% vs. 28%, *P* = 0.004). Both L+ (HR = 2.237, 95% CI: 1.178–4.247; *P* = 0.014) and G+ (HR = 1.872, 95% CI: 1.040–3.371; *P* = 0.037) independently predicted MACCEs after adjusting for age, NYHA class III–IV, LVOT obstruction and LVEF, adjusting for age, NYHA class III–IV, LVOT obstruction and LVEF. MACCE rates escalated across subgroups: G−/L− (22%), G+/L− (39%), G−/L+ (41%), and G+/L+ (63%) (*P* = 0.004). Among 89 patients with LVEF ≥150%, G+/L+ had the highest MACCE incidence (80% vs. 17% in G−/L−, *P* < 0.001).

**Conclusion:**

The combined assessment of genotype and late gadolinium enhancement significantly enhances risk stratification and prognosis prediction in hypertrophic cardiomyopathy patients, including those with preserved left ventricular ejection fraction, providing valuable insights for clinical decision-making.

## 1 Introduction

Hypertrophic cardiomyopathy (HCM) is a heritable, relatively common, and increasingly recognised cardiac disorder that is characterised by asymptomatic left ventricular (LV) hypertrophy (1). It is an important cause of sudden cardiac death, progressive heart failure, and atrial fibrillation (AF) (2, 3). The prevalence estimates are 1:500 based on the disease phenotype recognised by echocardiography (4), but approximately 1:200 when familial occurrence and contemporary diagnostic imaging are also considered (5). Up to 60% of HCM patients have pathogenic variants, including greater than 1,400 encoded sarcomere protein mutations in at least 14 genes (6, 7).

The variability between genotypes and phenotypes is much greater than consistency, and different mutations are found between different groups of people and different families. Therefore, the controversy on the relationship between genetic variation and phenotypic expression of HCM remains. This complexity presents challenges in risk stratification and individualized patient management.

The progress of genotype analysis technology and clinical evaluation tools, such as cardiac magnetic resonance (CMR) imaging, allows for precise information on left ventricular morphology (8) and provides information on the number and pattern of diffuse late gadolinium enhancement (LGE) in HCM (9). New methods are needed to evaluate genotype-phenotype correlation and risk assessment of HCM.

The present study investigated the combined effects of genetic variants and imaging phenotypic characteristics on prognosis in patients with HCM. Therefore, we aimed to evaluate whether combining genetic variant classification and LGE on CMR can improve prognostic stratification in patients with HCM.

## 2 Materials and methods

### 2.1 Subjects and design of the study

This study was a single-centre observational retrospective study on subjects with HCM recruited from the First People's Hospital of Yunnan Province, China.

HCM diagnosis was defined as a hypertrophied and non-dilated LV (wall thickness ≥15 mm) by cardiac magnetic resonance (CMR) in the absence of another cardiac or systemic disease that could likely produce a similar magnitude of hypertrophy (10).

We excluded patients with uncontrolled hypertension (blood pressure levels ≥140/90 mmHg), severe valvular heart disease (especially aortic valve stenosis), infiltrative cardiomyopathies, uncontrolled ventricular arrhythmias, other concomitant systemic diseases, and contraindications to CMR. Baseline demographic and clinical data were collected from clinical records, including age at first visit, sex, body mass index (BMI), cardiovascular risk factors, such as the presence of hypertension or diabetes, and clinical manifestations. Dyspnoea was classified using the New York Heart Association (NYHA) functional classification. CMR, transthoracic echocardiography, and 24-h electrocardiogram monitoring were performed.

We investigated clinical outcomes using medical record review. Major adverse cardiac and cerebrovascular events (MACCEs) were defined as cardiac death, progressive heart failure, sustained ventricular tachycardia/ventricular fibrillation (VT/VF), ICDs implantation, stroke, syncope, and atrial fibrillation (AF). Progressive heart failure was defined as follows: (1) Symptomatic worsening: An increase of ≥1 class in the NYHA functional classification. (2) Objective deterioration: Hospitalization necessitating intravenous diuretics or inotropes, or a decline in LVEF by ≥10% as assessed by echocardiography. (3) Biomarker evidence: A >2-fold increase in NT-proBNP levels from baseline. ICDs implantation was then tracked as an outcome subsequent to VT/VF occurrence.

### 2.2 Genetic testing and data analysis

Whole-exome sequencing (WES) was performed from blood samples in 135 HCM patients. Genomic DNA was isolated from blood samples. Products were purified using the AMPure XP system (Beckman Coulter, Beverly, USA), and DNA concentration was measured using a Qubit®3.0 Fluorometer (Invitrogen, USA). Libraries were analysed for size distribution using NGS3K/Calliper and quantified using real-time PCR (3 nM). The DNA library was sequenced on Illumina for paired-end 150 bp reads.

The exome sequences were efficiently enriched from 0.4 μg genomic DNA using an Agilent liquid capture system (Agilent SureSelect Human All Exon V6) according to the manufacturer's protocol. The clustering of the index-coded samples was performed on a cBot Cluster Generation System using an Illumina PE Cluster Kit (Illumina, USA) according to the manufacturer's instructions. Valid sequencing data were mapped to a reference genome (GRCh37/hg19/GRCh38) using BurrowsWheeler Aligner (BWA) software (11) to obtain the original mapping results in BAM format. SAMtools (12) mpileup and bcftools were used to perform variant calling and identify single nucleotide polymorphisms and indels. Control-FREEC (13) was used to perform copy number variation detection.

ANNOVAR was used to annotate the VCF (Variant Call Format) file obtained in the previous step (14). This annotation process provided detailed information on variant position, variant type, and conservation predictions, utilizing multiple databases including dbSNP, 1,000 Genomes, gnomAD, and HGMD. To identify potential pathogenic mutations, we focused on variants within a subset of cardiomyopathy/channel disease-related genes. The functional impact of these variants was further evaluated using in silico prediction tools, including PolyPhen-2 (15), SIFT (16), and MutationTaster (17), to distinguish putative pathogenic mutations from neutral variants. Finally, the variants identified by whole-exome sequencing (WES) were validated through PCR amplification of the corresponding exons, followed by confirmation using Sanger sequencing.

After systematic review by cardiovascular genetics experts, the genetic variants were centrally classified as pathogenic (P), likely pathogenic (LP), and unknown significance (VUS) according to the ACMG criteria (18, 19).

### 2.3 Cardiac magnetic resonance (CMR) imaging

CMR imaging was performed using a 1.5-T system (Siemens, Area, Germany). Breath-hold electrocardiography-gated cine steady-state free precession images were collected in short-axis slices and standard 2-, 3-, and 4-chamber long-axis directions (20). LGE on CMR was performed 10 min after the injection of 0.15 mmol/kg bodyweight gadobutrol (Gadovist®; Bayer Healthcare, Berlin, Germany) using a multi-shot, turbo field echo, breath-hold sequence with a phase-sensitive inversion recovery method, and images were obtained in the same views as the cine images.

Two CMR experts at the CMR core laboratory who were blinded to genotype and outcomes centrally evaluated cine and LGE images using cvi42 CircleCVI software. When disagreement occurred, the most experienced expert opinion prevailed. LV volume, mass, and LVEF were quantitatively measured from the stack of short-axis cine images using standard techniques (21). The presence and location of hyper-enhanced tissue on LGE was determined by visual inspection. The extent of LGE was qualitatively evaluated through visual assessment and recorded as either present (L+) or absent (L−). Point-like gadolinium delayed enhancement was categorized as L−. No quantitative analysis of LGE extent was performed.

### 2.4 2D echocardiography

Standard two-dimensional (2D) echocardiographic images were obtained with standard methods using a commercially available system (Vivid E9, GE Medical System, Norway). The probe frequency was 2.0–3.5 MHz. Multi-slice (left ventricular length, short axis, and infrarapier four lumen sections) and multiple body surface positions (apical four lumen) scanning were performed. Left ventricular size and wall thickness were conventionally measured by M-mode echocardiography using the 2005 American Society of Echocardiography (ASE) guidelines and standards (22). Left ventricular ejection fraction (LVEF) was estimated using the modified Simpson's method from apical imaging planes. Left ventricular outflow tract (LVOT) and mid-ventricular (MV) obstruction were defined as a peak pressure gradient >30 mmHg under resting conditions (23).

HCM was classified into four types according to the pattern and degree of LV hypertrophy using the following criteria: (i) hypertrophic obstructive cardiomyopathy (HOCM); (ii) septal ventricular hypertrophy (SVH); (iii) right ventricular hypertrophy (RVH); and (iv) apical hypertrophy alone (ApHCM).

### 2.5 Follow-ups

All patients were scheduled to undergo a review every 3–6 months at the First People's Hospital of Yunnan Province.

The follow-up for each patient was calculated from the date of their CMR study to the occurrence of any MACCEs, death from another cause, or the date of their most recent evaluation. Detailed information was obtained, including the current survival and clinical status (e.g., symptoms and NYHA classes), whether and when major adverse cardiac events and MACCEs had occurred, and any current treatments that patients were receiving.

### 2.6 Statistical analysis

Continuous variables are presented as the means ± standard deviation (SD). Student's *t*-test, the Mann–Whitney test, ANOVA, or the Kruskal–Wallis test was performed to test for differences in continuous variables. Comparisons between non-continuous categorical variables were performed using the chi-squared test or Fisher's exact test. Multivariable Cox proportional hazards models were constructed to adjust for clinically relevant confounders including age, NYHA class III–IV, LVOT obstruction, LVEF, LGE status, and genotype. The cumulative probability of an event on follow-up was estimated according to the Kaplan–Meier method and compared using the log-rank test. Data were analysed using R software version 4.2. All statistical tests were 2-tailed, and *P* < 0.05 was considered significant.

## 3 Results

### 3.1 Baseline patient characteristics

Between March 2018 and February 2024, a total of 144 unrelated consecutive subjects with HCM were included in this study. Six patients with absent and 3 patients with non-compaction were excluded. A total of 135 patients were finally analysed, with a median follow-up of 15 months (interquartile range 9–36). The characteristics of the patients are presented in Table 1. Myocardial scarring was present in 54 (40%) patients (L+), and 81 (60%) patients did not show LGE on CMR (L−). Male sex predominated (79%), and the mean age at diagnosis was 51.59 ± 11.10 years.

**Table 1:** Clinical characteristics of HCM patients: demographics, ECG, laboratory tests, and genetic mutations.

Variable	Total (*n* = 135)	L+ (*n* = 54)	L− (*n* = 81)	*P*-value
Demographics				
Sex, male (*n*)	107 (0.79)	42 (0.78)	65 (0.80)	0.729
Age (years, mean ± SD)	52.43 ± 11.35	52.57 ± 11.61	52.33 ± 11.25	0.904
Age of first visit (years, mean ± SD)	51.59 ± 11.10	50.65 ± 10.92	52.22 ± 11.23	0.421
BMI (kg/m^2^, mean ± SD)	23.5 ± 2.62	23.175 (20.93, 24.7275)	23.84 (22.2, 24.94)	0.243*
NYHA grade, III–IV (*n*)	29 (0.21)	20 (0.37)	9 (0.11)	<0.001
Previous HF admission (*n*)	20 (0.15)	14 (0.26)	6 (0.07)	0.003
Previous stroke (*n*)	1 (0.01)	0 (0.00)	1 (0.01)	1**
Previous VT or VF (*n*)	5 (0.04)	3 (0.06)	2 (0.02)	0.389**
Previous syncope (*n*)	8 (0.06)	4 (0.07)	4 (0.05)	0.713**
Previous AF (*n*)	14 (0.10)	9 (0.17)	5 (0.06)	0.05
Previous ICD (*n*)	1 (0.01)	1 (0.02)	0 (0.00)	0.4**
Diabetes (*n*)	2 (0.01)	1 (0.02)	1 (0.01)	1**
Hypertension (*n*)	20 (0.15)	15 (0.28)	5 (0.06)	<0.001
Baseline ECG				
AF (*n*)	20 (0.15)	8 (0.15)	12 (0.15)	1**
LBBB (*n*)	8 (0.06)	7 (0.13)	1 (0.01)	0.007**
QRS (mm, mean ± SD)	101.43 ± 20.12	96 (88, 114)	100 (88, 112)	0.248*
Abnormal T-wave inversion (*n*)	64 (0.47)	20 (0.37)	44 (0.54)	0.049
Pathological Q wave (*n*)	23 (0.17)	10 (0.19)	13 (0.16)	0.709
Laboratory tests				
NT-proBNP (ng/ml, mean ± SD)	2,720.28 ± 4,642.01	1,139 (342.25, 2,733.18)	754 (279, 2,711.24)	0.318
cTnT (ng/ml, mean ± SD)	1.01 ± 6.17	0.0195 (0.00075, 0.16)	0.03 (0.007, 0.20)	0.596
Mutant gene				
Mutant gene (*n*)	50 (0.37)	27 (0.50)	23 (0.28)	0.006

AF, atrial fibrillation; BMI, body mass index; cTnT, cardiac troponin T; ECG, electrocardiogram; ICD, implantable cardioverter-defibrillator; LBBB, left bundle branch block; NT-proBNP, N-terminal pro-brain natriuretic peptide; NYHA, New York Heart Association; VT, ventricular tachycardia; VF, ventricular fibrillation.

* Indicates the Mann–Whitney *U*-test was used.

** Indicates the Fisher's exact probability test was used.

Most patients were in NYHA class I or II (79%) at baseline, and patients with LGE had worse NYHA class (III–IV) (37% vs. 11%, *P* < 0.001) and higher rates of heart failure hospitalisation (26% vs. 7%, *P* = 0.003) compared to patients without LGE. There were no significant differences in the patient history of syncope, VT or VF. Patients with LGE had a higher rate of gene mutation (50% vs. 28%, *P* = 0.004).

LGE patients showed a higher rate of LBBB (13% vs. 1%, *P* = 0.007) and a lower rate of abnormal T-wave inversion (37% vs. 54%, *P* = 0.049). Other ECG findings, such as atrial fibrillation, pathological Q wave or the width of QRS, were similar. Clinical characteristics according to sex are provided in Supplementary Table S1.

### 3.2 Imaging characteristics by LGE status

Echocardiography and CMR characteristics for patients with and without LGE are shown in Table 2.

**Table 2:** Imaging features of HCM patients: echocardiographic and MRI parameters.

Variable	Total (*n* = 135)	L+ (*n* = 54)	L− (*n* = 81)	*P*-value
Baseline echocardiogram				
MWT (mm, mean ± SD)	17.78 ± 4.57	17.18 ± 4.66	18.18 ± 4.49	0.213
MWT ≥20 mm (*n*)	31 (0.23)	9 (0.17)	22 (0.27)	0.156
LA (mm, mean ± SD)	38.76 ± 6.79	39.22 ± 6.50	38.46 ± 7.01	0.528
RV (mm, mean ± SD)	20.33 ± 3.02	20.44 ± 3.56	20.25 ± 2.62	0.715
IVS (mm, mean ± SD)	16.69 ± 4.32	16 (14, 19)	17 (16, 22)	0.104*
LVPWT (mm, mean ± SD)	11.04 ± 2.17	10.68 ± 1.94	16 (13.6, 18.00)	0.116*
HOCM (*n*)	29 (0.21)	11 (0.20)	18 (0.22)	0.797
RVH (*n*)	1 (0.01)	1 (0.02)	0 (0.00)	0.4**
SVH (*n*)	120 (0.89)	47 (0.87)	73 (0.90)	0.576
ApHCM (*n*)	16 (0.12)	8 (0.15)	8 (0.10)	0.384
MRI baseline				
LVEF (%, mean ± SD)	52.79 ± 15.25	55 (39.75, 62)	58 (48, 65.50)	0.012*
LVEDV (ml, mean ± SD)	147.56 ± 55.5	135 (125.25, 213)	126 (106, 155.50)	0.004*
LVESV (ml, mean ± SD)	74.79 ± 52.33	64 (46.75, 114)	47 (39.50, 64.50)	0.003*
LVM (g, mean ± SD)	175.15 ± 62.16	173.71 ± 58.89	176.11 ± 64.59	0.827
CO (L/min, mean ± SD)	5.14 ± 1.58	5.04 ± 1.44	5.2 ± 1.67	0.566
CI (L/min/m2, mean ± SD)	2.92 ± 0.72	2.85 ± 0.72	2.97 ± 0.73	0.359
LVEF ≤50% (*n*)	46 (0.34)	23 (0.43)	23 (0.28)	0.062

ApHCM, apical ventricular hypertrophy cardiomyopathy; CI, cardiac index; CO, cardiac output; HOCM, hypertrophic obstructive cardiomyopathy; IVS, interventricular septum thickness; LA, left atrial; LGE, late gadolinium enhancement; LVEDV, left ventricular end-diastolic volume; LVEF, left ventricular ejection fraction; LVESV, left ventricular end-systolic volume; LVPWT, left ventricular posterior wall thickness; LVM, left ventricular mass; MRI, magnetic resonance imaging; MWT, maximal wall thickness; PASP, pulmonary artery systolic pressure; RV, right ventricular; RVH, right ventricular hypertrophy SVH, septal ventricular hypertrophy.

* Indicates the Mann–Whitney *U*-test was used.

** Indicates the Fisher's exact probability test was used.

The average maximal LV wall thickness was 17.78 ± 4.57 mm, and the mean interventricular septum (IVS) thickness was 16.69 ± 4.32 mm. Septal hypertrophy (89%) was the most common morphological subtype, but no significant difference was detected between the groups. LVOT obstruction was found in 29 patients (21%), with no significant difference between the groups.

The mean LV mass was 175.15 ± 62.16 g. Patients with LGE had a higher LVEDV compared to patients without LGE (median: 135, IQR: 125.25–213.00 vs. median: 126, IQR: 106.00–155.50, *P* = 0.004). The mean LVEF at CMR was 52.79 ± 15.25%, and 45 (34%) patients had an LVEF ≤50%. Patients with LGE had lower LVEF (median: 55%, IQR: 39.75%–62% vs. median: 58%, IQR: 48%–65.5%, *P* = 0.012).

### 3.3 Genetic variant profile of HCM patients

Fifty (37.03%) of the 135 HCM patients carried at least one P/LP variant that was associated with HCM. One patient exhibited two mutations involving HCM-related genes. Within the G+ group, most of the mutations mapped to sarcomeric loci (82%, *n* = 41). Among all of the sarcomere gene mutations detected in this study, mutations in MYH7 (46%) were the most common, followed by mutations in MYBPC3 (16%), TNNT2 (8%), TNNI3 (6%) and MYL3 (4%). The distribution of identified mutations for each analysed gene is shown in Supplementary Table S2.

The clinical characteristics of G+ and G− patients are provided in Supplementary Table S3. There were no significant differences in the average age or sex ratio between the two groups. The presence of CV risk factors, such as hypertension and diabetes, also did not differ. Both groups exhibited similar baseline echocardiographic LVEF and maximum wall thickness, a similar percentage of patients with LVEF ≤50% and similar NYHA class, but patients with G+ had lower CO on CMR (4.91 ± 1.35 vs. 5.34 ± 1.74, *P* = 0.112). G+ patients had a higher rate of abnormal T-wave inversion (62% vs. 39%, *P* = 0.009).

### 3.4 Clinical outcomes and predictors of MACCEs

During the follow-up period, 50 (37%) patients experienced MACCEs, including 4 (3%) cases of cardio-death, 19 (14%) cases of HF aggravation, 8 (7%) cases of sustained VT/VF, 3 (2%) cases of stroke, 13 (10%) cases of syncope, and 14 (10%) cases of new-onset AF (Supplementary Table S4). In contrast, L+ patients showed a higher incidence of MACCEs than L− patients (52% vs. 27%, *P* = 0.004). Patients with LGE were more likely to experience sustained VT or VF, and a significant difference was detected between the groups (11% vs. 2%, *P* = 0.03).

Patients with gene mutations had a higher incidence of MACCEs (52% vs. 28%, *P* = 0.006). And they were also more likely to develop sustained VT or VF (*P* = 0.022), which led to a higher frequency of ICD implantation (*P* = 0.143) (Supplementary Table S5).

However, we did not observe a significant association between MACCEs and sex (*P* = 0.248) or LVEF (<50% and ≥50%) (*P* = 0.208) (Supplementary Tables S6, S7).

Univariate analysis (Model 1) confirmed that both LGE positivity (L+) and the presence of pathogenic genetic variants (G+) were significant predictors of MACCEs. Specifically, patients with L+ exhibited a 2.884-fold increased risk of MACCEs (HR = 2.884, 95% CI: 1.636–5.086, *P* < 0.001), while the presence of pathogenic genetic variants was associated with a 2.123-fold elevated risk (HR = 2.123, 95% CI: 1.216–3.704, *P* = 0.008). To account for potential confounders, multivariable Cox regression (Model 2) was performed, adjusting for age, NYHA class III–IV, LVOT obstruction, LVEF, LGE status, and genotype. As shown in Table 3, both L+ (HR = 2.237, 95% CI: 1.178–4.247; *P* = 0.014) and G+ (HR = 1.872, 95% CI: 1.040–3.371; *P* = 0.037) remained independent predictors of MACCEs after adjustment. Stratification by combined genotype/LGE groups (Model 3) revealed a pronounced risk gradient (*P* = 0.003): (1) G+/L+: A 4.25-fold higher MACCE risk compared to G−/L− (HR = 4.246, 95% CI: 1.965–9.171, *P* < 0.001); (2) G−/L+: A 2.7-fold increased risk (HR = 2.706, 95% CI: 1.165–6.288, *P* = 0.021); (3) G+/L−: Borderline significance (HR = 2.348, 95% CI: 0.979–5.635, *P* = 0.056).

**Table 3:** Cox regression analysis for MACCEs.

Variable	*P*-value	HR	95% CI
Model 1 Univariate analysis			
L (+)	<0.001	2.884	1.636–5.086
G (+)	0.008	2.123	1.216–3.704
Model 2 Multivariate analysis			
Age (years)	0.970	1.001	0.975–1.027
NYHA, III–IV	0.084	1.812	0.924–3.555
LVOT	0.054	1.895	0.988–3.635
LVEF (%)	0.674	1.004	0.984–1.025
L (+)	0.014	2.237	1.178–4.247
G (+)	0.037	1.872	1.040–3.371
Model 3 Multivariate analysis			
Age (years)	0.983	1.000	0.974–1.026
NYHA, III–IV	0.077	1.853	0.936–3.669
LVOT	0.044	1.970	1.019–3.809
LVEF (%)	0.666	1.004	0.985–1.025
Combination of genotype and LGE	0.003		
G(−)/L(+)	0.021	2.706	1.165–6.288
G(+)/L(−)	0.056	2.348	0.979–5.635
G(+)/L(+)	<0.001	4.246	1.965–9.171

Model 1 (Univariate analysis): Independently assess the LGE status, and genotype with MACCEs.

Model 2 (Multivariate analysis): After adjusting for confounder: age, NYHA class III–IV, LVOT obstruction, LVEF, LGE status, and genotype.

Model 3 (Combined analysis): Evaluate the interaction effect between genotype and LGE status.

**Figure 1:**
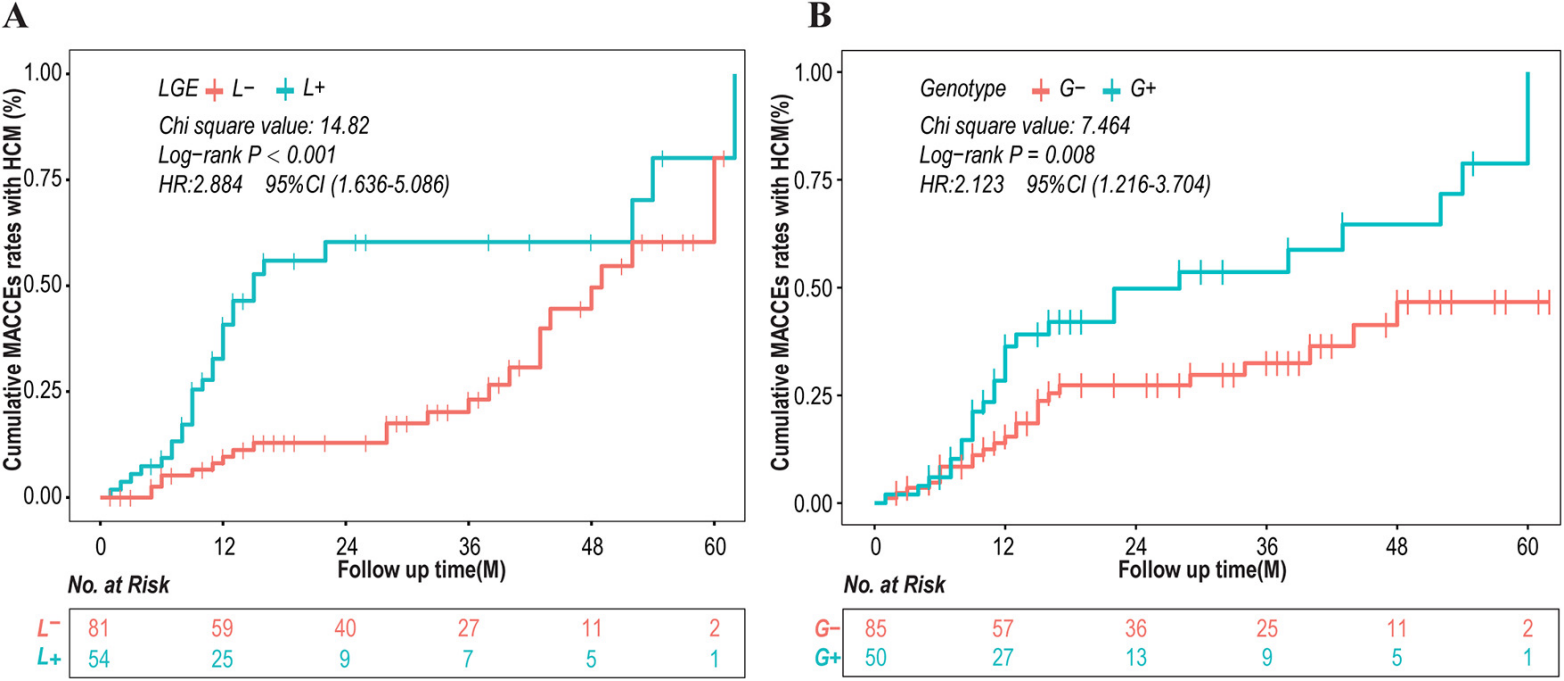
Kaplan–Meier curves of cumulative MACCEs according to presence of late gadolinium enhancement (LGE) and genotype. CI, confidence interval; HCM, hypertrophic cardiomyopathy; HR, hazard ratio; MACCEs, major adverse cardiac and cerebrovascular events.

Survival analysis found that the rates of MACCEs were associated with L+ (ꭓ^2^ = 14.82, log-rank test *P* < 0.001, HR = 2.884, 95% CI: 1.636–5.086) and gene mutation (ꭓ^2^ = 7.464, *P* = 0.008, HR = 2.123, 95% CI: 1.216–3.704). Kaplan–Meier curves of cumulative MACCEs according to the presence of LGE and genotype are shown in Figure 1.

### 3.5 Combined genotype and LGE stratification

Classification of patients based on genotype (G+/G−) and LGE (L+/L−) revealed progressively increasing events across G−/L−, G+/L−, G−/L+ and G+/L+ groups. The clinical characteristics of the patients included in each group are shown in Supplementary Table S8. The following incidence of MACCEs per group was observed in Table 4: 63% in G+/L+, 39% in G+/L−, 41% in G−/L+, and 22% in G−/L−. The difference was statistically significant (*P* = 0.004). Interventions, such as ICD implantation, were more frequently performed in L+/G+ patients (*P* = 0.033).

**Table 4:** Outcome and events of HCM patients according to combination of genotype and LGE.

Clinical events	Total (*n* = 135)	G+/L+ (*n* = 27)	G+/L− (*n* = 23)	G−/L+ (*n* = 27)	G−/L− (*n* = 58)	*P*-value
MACCEs (*n*)	50 (0.37)	17 (0.63)	9 (0.39)	11 (0.41)	13 (0.22)	0.004
Cardio-death (*n*)	4 (0.03)	1 (0.04)	1 (0.04)	1 (0.04)	1 (0.02)	0.904*
Progress HF (*n*)	19 (0.14)	5 (0.19)	6 (0.26)	3 (0.11)	5 (0.09)	0.186*
VT or VF (*n*)	8 (0.06)	4 (0.15)	2 (0.09)	2 (0.07)	0 (0.00)	0.048*
Stroke (*n*)	3 (0.02)	2 (0.07)	0 (0.00)	0 (0.00)	1 (0.02)	0.208*
Syncope (*n*)	13 (0.10)	4 (0.15)	3 (0.13)	3 (0.11)	3 (0.05)	0.469*
AF (*n*)	14 (0.10)	5 (0.15)	2 (0.07)	3 (0.14)	4 (0.08)	0.429*
ICDs implantation (*n*)	4 (0.03)	3 (0.12)	0 (0.00)	1 (0.04)	0 (0.00)	0.033*

Values are *n* (%).

AF, atrial fibrillation; ICD, implantable cardioverter-defibrillator; MACCE, major adverse cardiac and cerebrovascular events; VT, ventricular tachycardia; VF, ventricular fibrillation.

* Indicates the Fisher's exact probability test was used.

**Figure 2:**
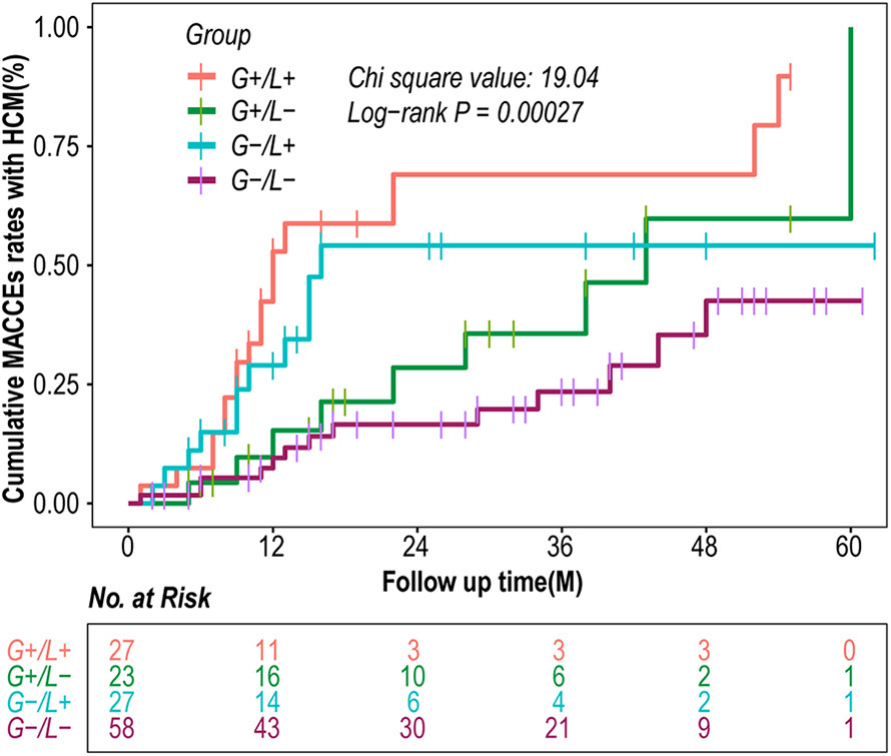
Kaplan–meier curves of MACCEs according to combination of genotype and LGE. CI, confidence interval; HCM, hypertrophic cardiomyopathy; HR, hazard ratio; MACCEs, major adverse cardiac and cerebrovascular events.

Survival analysis showed a gradual increase in events in the G−/L−, G+/L−, G −/L+, and G+/L+ groups (ꭓ*^2^* = 19.04, *P* = 0.00027) (Figure 2).

Patients with HCM and LVEF ≥50% (*n* = 89, 65.93%) showed different outcomes during follow-up (Table 5). Specifically, the incidence of MACCEs per group was 80% in G+/L+, 46% in G+/L−, 25% in G−/L+, and 18% in G−/L−, and the difference was statistically significant (*P* < 0.001).

**Table 5:** Outcome and events of HCM patients with LVEF ≥50% according to combination of genotype and LGE.

Clinical events	Total (*n* = 89)	G+/L+ (*n* = 15)	G+/L− (*n* = 13)	G−/L+ (*n* = 16)	G−/L− (*n* = 45)	*P-*value
MACCEs (*n*)	30 (0.33)	12 (0.80)	6 (0.46)	4 (0.25)	8 (0.18)	<0.001
Cardio-death (*n*)	2 (0.02)	1 (0.07)	0 (0.00)	0 (0.00)	1 (0.02)	0.572*
Progress HF (*n*)	9 (0.10)	4 (0.27)	3 (0.23)	1 (0.06)	1 (0.02)	0.016*
VT or VF (*n*)	4 (0.06)	3 (0.20)	0 (0.00)	1 (0.06)	0 (0.00)	0.010*
Stroke (*n*)	2 (0.02)	2 (0.13)	0 (0.00)	0 (0.00)	0 (0.00)	0.018*
Syncope (*n*)	9 (0.10)	3 (0.20)	1 (0.08)	2 (0.13)	3 (0.07)	0.496*
AF (*n*)	8 (0.09)	2 (0.13)	2 (0.15)	1 (0.06)	3 (0.07)	0.696*
ICDs implantation (*n*)	3 (0.03)	2 (0.13)	0 (0.00)	1 (0.06)	0 (0.00)	0.072*

Values are *n* (%).

AF, atrial fibrillation; ICD, implantable cardioverter-defibrillator; MACCE, major adverse cardiac and cerebrovascular events; VT, ventricular tachycardia; VF, ventricular fibrillation.

* Indicates the Fisher's exact probability test was used.

## 4 Discussion

The present single-centre observational retrospective study investigated the distribution of genetic variations and related differences in clinical and imaging phenotypic characteristics in patients with HCM. Previous studies showed that the presence of LGE and P/LP genetic variants were associated with worse prognosis during mid-term follow-up. The classification of patients based on genotype (G+/G−) and LGE (L+/L−) revealed progressively increasing events in the G−/L−, G+/L−, G−/L+ and G+/L+ groups and optimised the prediction of MACCEs, even in patients with LVEF ≥150%. Patients without LGE or genetic variants exhibited a favorable prognosis, with a very low incidence of MACCEs during follow-up.

Genetic testing for HCM is used clinically in the form of targeted exonic sequencing of known pathogenic variants (24). Over 27 genes and 1,400 HCM-related locations have been detected (25). Mutations in the known sarcomere protein gene were previously detected in approximately 35%–65% of HCM patients (26). Our study found that 37.03% of patients with HCM had identified variant mutations using WES. Most variants were located at the sarcomeric loci, which accounted for 82% of variant detected cases, and most variants were present in MYH7 (46%) and MYBPC3 (16%), followed by other variants of TNNT2 (8%), TNNI3 (6%) and MYL3 (4%). The distribution of variation based on gene location in our study was similar to previous studies on sarcomeric variants (7, 27).

There have been various outcomes on the prognostic significance of genotypic analysis in HCM. Some studies showed that patients with positive sarcomere variants had a poor prognosis, including an increased risk of heart disease death, progression to NYHA class III or IV HF, and stroke (28), but other studies showed that gene mutations had no significance in the future (29). In our cohort, patients with identified mutations had an elevated risk of MACCEs, particularly in the presence of myocardial fibrosis and VT/VF. Because there are few reports on the genotype-phenotype relationship of HCM based on CMR, more active research may be performed in the future (30).

CMR's high spatial and temporal resolution enables precise assessment of myocardial morphology and ventricular function (31), with LGE quantification emerging as a key prognostic tool in HCM (32). Consistent with prior study (33), we observed that LGE was associated with worse NYHA functional class (III/IV), higher heart failure hospitalization rates, and increased MACCEs. Notably, LGE-positive patients also had a higher incidence of sustained VT/VF. However, unlike other study (34), we found no significant sex-based differences in LGE prevalence, genetic diagnosis, or clinical outcomes.

Our study highlights the utility of CMR in genotype-phenotype analysis, demonstrating a strong association between genetic mutations and LGE. This aligns with previous findings that LGE is more prevalent in genetically positive patients (35).

Another finding of our study is that the combination of LGE and genotype improves prognosis prediction in HCM patients. Patients with LGE and/or positive genotypes exhibit significantly worse prognosis compared to patients without LGE on CMR and G−. It's interesting that the prognoses of the LGE-positive but no genetic abnormality group and the LGE-negative but with genetic abnormality group are comparable. We hypothesize that the increased risk of MACCEs in the G−/L+ group is primarily attributable to advanced myocardial disease, including fibrosis and ventricular dysfunction, whereas the elevated risk in the G+/L− group may be attributed to an inherent genetic predisposition, despite the preservation of myocardial structure at present. Notably, the LGE/genotype classification was also useful in patients with HCM and LVEF ≥150%. Current guidelines recommend HCM with LV systolic dysfunction (EF <50% by echocardiography or CMR imaging) for risk stratification of SCD (3). Among the 89 patients with LVEF ≥150% in the study (65.93%), patients who had LGE and who were G+ had higher MACCEs occurrence than patients who were L−/G−, with an absolute rate of MACCEs of 80% in patients with L+/G+ compared to18% in patients who were L−/G−. Therefore, genotype and LGE grouping in patients with HCM and LVEF >50% also resulted in improved MACCEs prediction.

Although our findings must be validated in other cohorts and ideally tested in prospective clinical trials, the combination of LGE and genotype improves prognosis prediction in patients with HCM.

Some limitations of this investigation must be noted. This study was an observational retrospective study, and our findings must be replicated in validation cohorts. A longer follow-up period would be necessary to fully assess the sustainability of the observed effects. The number of patients included was relatively small, and the frequency of adverse events was low, which resulting in limited statistical power. Only patients who were able to undergo CMR imaging and genetic testing were included. Furthermore, a larger number of genes and loci remain unexamined. Consequently, the potential for selection bias cannot be excluded. Additionally, we note that extensive LGE quantification (≥15% of LV mass) has been recognized as the indication for primary ICD in both 2022 ESC and 2020 AHA/ACC models (3, 36). Future studies are needed to explore the combination of LGE quantification and genetic phenotype, in order to better evaluate the prognosis of HCM patients.

## 5 Conclusions

We found that LGE correlated with genetic phenotype, and the combination of scar assessment with CMR and genotyping was a more reliable predictor of cardiovascular events in patients with HCM. Genotype and LGE grouping in patients with HCM and LVEF ≥50% also resulted in improved MACCEs prediction.

## Data Availability

The original contributions presented in the study are publicly available. The total variation data have been deposited in the CliVar submission (SUB14807938) (https://submit.ncbi.nlm.nih.gov/subs/clinvar_org/SUB14807938/overview).
