# Supplementary material for: The associations among genetic features, late gadolinium enhancement and prognosis in hypertrophic cardiomyopathy
Source: Front Cardiovasc Med. 2025 Jul 7;12:1597405. doi: 10.3389/fcvm.2025.1597405 (PMC12277264; doi:10.3389/fcvm.2025.1597405)
Supplement: Supplementary file 1 [file Datasheet1.pdf]

SUPPLEMENTAL MATERIAL

The associations among genetic features, late gadolinium enhancement and prognosis in hypertrophic cardiomyopathy

Supplemental Tables

Table S1. Characteristics of HCM patients according to sex.

Table S2. 20 sarcomeric and non-sarcomeric genes of hypertrophic cardiomyopathy patients.

Table S3. Characteristics of HCM patients according to mutant gene.

Table S4. Outcome and events of HCM patients according to LG.

Table S5. Outcome and events of HCM patients according to gene.

Table S6. Outcome and events of HCM patients according to sex.

Table S7. Outcome and events of HCM patients according to LVEF of CMR.

Table S8. Characteristics of HCM patients according to combination of genotype and LGE.

Table S1. Characteristics of HCM patients according to sex.

	total(n=135)	male(n=107)	female(n=28)	*P*-value
Demographics				
Age (years, mean ± SD)	52.43 ± 11.35	51.59 ± 10.22	55.64 ± 14.7	0.093
Age of first visit (years, mean ± SD)	51.59 ± 11.1	50.87 ± 9.91	54.36 ± 14.67	0.139
BMI (kg/m^2, mean ± SD)	23.5 ± 2.62	23.52 ± 2.64	23.42 ± 2.61	0.848
NYHA grade, III-IV(n)	29(0.21)	24(0.22)	5(0.18)	0.6
Previous heart failure admission (n)	20(0.15)	16(0.15)	4(0.14)	1*
Previous stroke (n)	1(0.01)	1(0.01)	0(0)	1*
Previous VT or VF (n)	5(0.04)	5(0.05)	0(0)	0.583*
Previous syncope (n)	8(0.06)	5(0.05)	3(0.11)	0.362*
Previous AF (n)	14(0.10)	14(0.11)	0(0)	1*
Previous ICD (n)	1(0.01)	1(0.01)	0(0)	1*
Diabetes (n)	2(0.01)	2(0.02)	0(0)	1*
Hypertension (n)	20(0.15)	16(0.15)	4(0.14)	1*
Baseline Echocardiogram				
Maximum wall thickness (mm, mean ± SD)	17.78±4.57	17.75 ± 4.65	17.91± 4.33	0.866
MWT≥20 mm (n)	31(0.23)	26(0.24)	5(0.18)	0.471
LA (mm, mean ± SD)	38.76 ± 6.79	39.22 ± 7.25	37.01 ± 4.32	0.127
RV (mm, mean ± SD)	20.33 ± 3.02	20.59 ± 3.15	19.33 ± 2.19	**0.048**
LVPW (mm, mean ± SD)	11.04 ± 2.17	10.94 ± 2.07	11.43 ± 2.52	0.282
HOCM (n)	29(0.21)	22(0.21)	7(0.25)	0.611
RVH (n)	1(0.01)	0(0)	1(0.04)	0.207*
SVH (n)	120(0.89)	93(0.87)	27(0.96)	0.195*
ApHCM (n)	16(0.12)	14(0.13)	2(0.07)	0.523*
MRI Baseline				
IVS (mm, mean ± SD)	18.16 ± 4.75	18.21 ± 4.72	17.96 ± 4.95	0.805
LVEF (%, mean ± SD)	52.79 ± 15.25	52.33±15.25	54.57±15.42	0.490
LVEDV (ml, mean ± SD)	147.56 ± 55.5	152±58.45	130.57±38.66	0.069
LVESV (ml, mean ± SD)	74.79 ± 52.33	78.06±54.91	62.32±39.37	0.157
LVM (g, mean ± SD)	175.15 ± 62.16	178.39 ± 58.71	162.79 ± 73.78	0.239
CO (L/min, mean ± SD)	5.14 ± 1.58	5.19 ± 1.61	4.93 ± 1.46	0.438
CI (L/min/m2, mean ± SD)	2.92 ± 0.72	2.93 ± 0.75	2.9 ± 0.64	0.867
LVEF ≤50% (n)	45(0.34)	37(0.36)	8(0.27)	0.392
LGE (n)	54(0.4)	42(0.39)	12(0.43)	0.729
Baseline ECG				
Sinus rhythm (n)	95(0.7)	76(0.71)	19(0.68)	0.744
AF (n)	20(0.15)	17(0.16)	3(0.11)	0.765*
LBBB (n)	8(0.06)	6(0.06)	2(0.07)	0.670*
QRS (mm, mean ± SD)	101.43 ± 20.12	102.26 ± 19.09	98.15 ± 23.9	0.344
Abnormal T-wave inversion (n)	64(0.47)	49(0.46)	15(0.54)	0.463
Pathological Q wave (n)	23(0.17)	21(0.2)	2(0.07)	0.16*
Laboratory Tests				
NT-proBNP (ng/ml, mean ± SD)	2720.28 ± 4642.01	2596.54 ± 4549.32	3193.12 ± 5039.72	0.547

CK-MB (ng/ml, mean ± SD)	6.36 ± 14.31	5.82 ± 13.89	8.44 ± 15.91	0.390
cTnT (ng/ml, mean ± SD)	1.01 ± 6.17	1.18 ± 6.9	0.37 ± 1.18	0.535
Mutant Gene				
Mutant gene (n)	50(0.37)	43(0.40)	19(0.68)	**0.009**

AF, atrial fibrillation; ApHCM, apical ventricular hypertrophy cardiomyopathy; BMI, body mass index; CI, cardiac index; CK-MB, creatine kinase MB; CO, cardiac output; cTnT, cardiac troponin T; ECG, electrocardiogram; HOCM, hypertrophic obstructive cardiomyopathy; ICD, implantable cardioverter-defibrillator; IVS, interventricular septum thickness; LA, left atrial; LBBB, left bundle branch block; LGE, late gadolinium enhancement; LVEDV, left ventricular end-diastolic volume; LVEF, left ventricular ejection fraction; LVESV, left ventricular end-systolic volume; LVPW, left ventricular posterior wall thickness; LVM, left ventricular mass; MRI, magnetic resonance imaging; MWT, maximal wall thickness; NT-proBNP, N-terminal pro-brain natriuretic peptide; NYHA, New York Heart Association; RV, right ventricular; RVH, right ventricular hypertrophy SVH, septal ventricular hypertrophy; VT, ventricular tachycardia; VF, ventricular fibrillation.

Table S2. 10 sarcomeric and non-sarcomeric genes of hypertrophic cardiomyopathy patients.

Case No.	Gene	Trancript	Nucleotide Change	Protein Change
2	*ALPK3*	NM_020778	c.4819C>T	p.Arg1607X
4	*MYH7*	NM_000257	c.730T>C	p.Phe244Leu
8	*MYH7*	NM_000257.2	c.3134G>A	p.Arg1045His
9	*TNNI3*	NM_000363	c.370G>C	p.Glu124Gln
13	*MYH7*	NM_000257.2	c.3134G>A	p.Arg1045His
17	*MYL3*	NM_000258	c.92G>A	p.Arg31His
19	*MYBPC3*	NM_000256.3	c.2450G>A	p.Arg817Gln
22	*MYH7*	NM_000257.2	c.161G>A	p.Arg54Gln
23	*MYH7*	NM_000257.2	c.1987C>T	p.Arg663Cys
24	*MYBPC3*	NM_000256.3	c.2450G>A	p.Arg817Gln
30	*TNNI3*	NM_000363.4	c.433C>G	p.Arg145Gly
31	*MYBPC3*	NM_001105206	c.3356C>G	p.Asp228Glu
32	*MYBPC3*	NM_000256.3	c.2992C>G	p.Gln998Glu
33	*MYH7*	NM_000257	c.2155C>T	p.Arg719Trp
35	*MYH7*	NM_000257.2	c.2155C>T	p.Arg719Trp
36	*FLNC*	NM_001127487	c.5369C>T	p.Thr1790Met
40	*TNNT2*	NM_001001432	c.839G>A	p.Arg280His
45	*TNNT2*	NM_001001432	c.839G>A	p.Arg280His
48	*TNNT2*	NM_001276345.1	c.887G>A	p.Arg296His
52	*MYH7*	NM_000257	c.730T>C	p.Phe244Leu
54	*MYH7*	NM_000257.2	c.1987C>T	p.Arg663Cys
57	*FLNC*	NM_001458	c.5278G>A	p.Gly1760Ser
59	*MYH7*	NM_000257.2	c.77C>T	p.Ala26Val
60	*MYH7*	NM_000257.2	c.77C>T	p.Ala26Val
63	*MYH7*	NM_000257.2	c.2654A>C	p.Asn885Thr
66	*MYL3*	NM_000258	c.92G>A	p.Arg31His
67	*MYH7*	NM_000257.2	c.1128C>A	p.Asp376Glu
74	*MYH7*	NM_000257.2	c.2572C>T	p.Arg858Cys
76	*MYH7*	NM_000257	c.2609G>A	p.Arg870His
80	*ACTN2*	NM_001103	c.947T>C	p.Met316Thr
81	*ACTN2*	NM_001103	c.1423G>A	p.Asp475Asn
83	*TNNI3*	NM_000363	c.433C>G	p.Arg145Gly
84	*MYBPC3*	NM_000256.3	c.706A>G	p.Ser236Gly
86	*TNNT2*	NM_001001432	c.740A>G	p.Lys247Arg
88	*MYH7*	NM_000257.2	c.2155C>T	p.Arg719Trp
90	*MYH7*	NM_000257	c.428G>A	p.Arg143Gln
92	*BAG3*	NM_004281	c.652C>T	p.Arg218W
93	*MYBPC3*	NM_000256	c.1224-10G>A	p.(?)
94	*MYBPC3*	NM_000256	c.2864_2865del	p.Pro955fs

95	*MYBPC3*	NM_000256.3	c.3624_3624delC	p.Pro1208fs
98	*MYH7*	NM_000257	c.428G>A	p.Arg143Gln
99	*TNNI3*	NM_000363	c.557G>A	p.Arg186Gln
103	*MYH7*	NM_000257	c.1208G>A	p.Arg403Gln
107	*MYH7*	NM_000257	c.1508G>A	p.Arg502Gln
108	*MYH7*	NM_000257	c.3548C>T	p.Arg1193Cys
115	*MYH7*	NM_000257	c.3134G>A	p.Arg1045His
119	*MYH7*	NM_000257	c.1231G>A	p.Val411Ile
122	*BAG3*	NM_004281	c.652C>T	p.Arg218W
129	*PRKAG2*	NM_001040633	c.*48T>C	p.(?)
135	*PRKAG2*	NM_016203.3	c.298G>A	p.Gly100Ser

Table S3. Characteristics of HCM patients according to genotype.

	total(n=135)	G+(n=50)	G− (n=85)	*P*-value
Demographics				
Sex, male (n)	107(0.79)	40(0.80)	67(0.79)	0.871
Age (years, mean ± SD)	52.43 ± 11.35	52.73 ± 12.25	52.18 ± 10.62	0.781
Age of first visit (years, mean ± SD)	51.59 ± 11.1	51.19 ± 11.66	51.93 ± 10.66	0.702
BMI (kg/m^2, mean ± SD)	23.5 ± 2.62	23.38 ± 2.71	23.60 ± 2.56	0.624
NYHA grade, III-IV(n)	29(0.21)	13(0.26)	16(0.19)	0.327
Previous heart failure admission (n)	20(0.15)	10(0.20)	10(0.12)	0.193
Previous stroke (n)	1(0.01)	0(0)	1(0.01)	1*
Previous VT or VF (n)	5(0.04)	3(0.06)	2(0.02)	0.359*
Previous syncope (n)	8(0.06)	5(0.10)	3(0.04)	0.146*
Previous AF (n)	14(0.10)	5(0. 10)	9(0.11)	0.914
Previous ICD (n)	1(0.01)	1(0.02)	0(0)	0.370*
Diabetes (n)	2(0.01)	1(0.02)	1(0.01)	1*
Hypertension (n)	20(0.15)	11(0.22)	9(0.11)	0.083
Baseline Echocardiogram				
MWT (mm, mean ± SD)	17.78±4.57	17.31 ± 4.59	18.19 ± 4.55	0.267
MWT≥20 mm (n)	31(0.23)	12(0.24)	19(0.22)	0.824
LA (mm, mean ± SD)	38.76 ± 6.79	38.57 ± 6.32	38.92 ± 7.21	0.766
RV (mm, mean ± SD)	20.33 ± 3.02	20.41 ± 3.38	20.26 ± 2.69	0.772
LVPW (mm, mean ± SD)	11.04 ± 2.17	11.22 ± 2.24	10.88 ± 2.12	0.363
HOCM (n)	29(0.21)	13(0.26)	16(0.19)	0.327
RVH (n)	1(0.01)	1(0.02)	0(0)	0.370*
SVH (n)	120(0.89)	43(0.86)	77(0.91)	0.413
ApHCM (n)	16(0.12)	8(0.16)	8(0.09)	0.253
MRI Baseline				
IVS (mm, mean ± SD)	18.16 ± 4.75	17.92 ± 4.48	18.37 ± 4.92	0.585
LVEF (%, mean ± SD)	52.79 ± 15.25	50.87 ± 14.45	54.42 ± 15.00	0.178
LVEDV (ml, mean ± SD)	147.56 ± 55.5	148.05 ± 62.48	148.23 ± 53.96	0.985
LVESV (ml, mean ± SD)	74.79 ± 52.33	79.37 ± 60.84	71.77 ± 47.99	0.419
LVM (g, mean ± SD)	175.15 ± 62.16	178.35 ± 66.05	172.44 ± 59.98	0.584
CO (L/min, mean ± SD)	5.14 ± 1.58	4.91 ± 1.35	5.34 ± 1.74	0.112
CI (L/min/m2, mean ± SD)	2.92 ± 0.72	2.82 ± 0.64	3.01 ± 0.78	0.128
LVEF ≤50% (n)	46(0.34)	22(0.44)	24(0.28)	0.062
LGE (n)	54(0.4)	27(0.54)	27(0.32)	0.067
Baseline ECG				
AF (n)	20(0.15)	8(0.16)	12(0.14)	0.766
LBBB (n)	8(0.06)	5(0.10)	3(0.04)	0.146*
QRS (mm, mean ± SD)	101.43 ± 20.12	102.84 ± 23.55	100.22 ± 16.71	0.455
Abnormal T-wave inversion (n)	64(0.47)	31(0.62)	33(0.39)	**0.009**
Pathological Q wave (n)	23(0.17)	9(0.18)	14(0.17)	0.819
Laboratory Tests				
NT-proBNP (ng/ml, mean ± SD)	2720.28 ± 4642.01	3749.36 ± 6288.45	1846.26 ± 2216.82	**0.017**
CK-MB (ng/ml, mean ± SD)	6.36 ± 14.31	10.08 ± 20.35	3.20 ± 2.80	**0.005**

cTnT (ng/ml, mean ± SD)	1.01 ± 6.17	1.88 ± 9.06	0.28 ± 0.46	0.168

AF, atrial fibrillation; ApHCM, apical ventricular hypertrophy cardiomyopathy; BMI, body mass index; CI, cardiac index; CK-MB, creatine kinase MB; CO, cardiac output; cTnT, cardiac troponin T; ECG, electrocardiogram; HOCM, hypertrophic obstructive cardiomyopathy; ICD, implantable cardioverter-defibrillator; IVS, interventricular septum thickness; LA, left atrial; LBBB, left bundle branch block; LGE, late gadolinium enhancement; LVEDV, left ventricular end-diastolic volume; LVEF, left ventricular ejection fraction; LVESV, left ventricular end-systolic volume; LVPW, left ventricular posterior wall thickness; LVM, left ventricular mass; MRI, magnetic resonance imaging; MWT, maximal wall thickness; NT-proBNP, N-terminal pro-brain natriuretic peptide; NYHA, New York Heart Association; RV, right ventricular; RVH, right ventricular hypertrophy SVH, septal ventricular hypertrophy; VT, ventricular tachycardia; VF, ventricular fibrillation.

Table S4. Outcome and events of HCM patients according to LGE.

**Clinical events**	**Total (n=135)**	**LGE+ (n=54)**	**LGE− (n=81)**	***P*-value**
MACCE events (n)	50(0.37)	28(0.52)	22(0.27)	**0.004**
Cardio-death (n)	4(0.03)	2(0.04)	2(0.02)	1*
Progress heart failure (n)	19(0.14)	8(0.15)	11(0.14)	0.84*
VT or VF (n)	8(0.07)	6(0.11)	2(0.02)	**0.03***
Stroke (n)	3(0.02)	2(0.04)	1(0.01)	0.564*
Syncope (n)	13(0.1)	7(0.13)	6(0.07)	0.284
AF (n)	14(0.1)	8(0.15)	6(0.07)	0.167
ICD Implanted (n)	4(0.03)	4(0.07)	0(0)	**0.024***

Values are n (%). * indicates the Fisher's exact probability test was used.
AF, atrial fibrillation; ICD, implantable cardioverter-defibrillator; MACCE, major adverse cardiac and cerebrovascular events; VT, ventricular tachycardia; VF, ventricular fibrillation.

Table S5. Outcome and events of HCM patients according to genotype.

**Clinical events**	**Total (n=135)**	**G+ (n=50)**	**G−(n=85)**	***P*-value**
MACCE events (n)	50(0.37)	26(0.52)	24(0.28)	**0.006**
Cardio-death (n)	4(0.03)	2(0.04)	2(0.02)	0.627*
Progress heart failure (n)	19(0.14)	11(0.22)	8(0.09)	0.042
VT or VF (n)	8(0.06)	6(0.12)	2(0.02)	**0.022***
Stroke (n)	3(0.02)	2(0.04)	1(0.01)	0.555*
Syncope (n)	13(0.1)	7(0.14)	6(0.07)	0.231
AF (n)	14(0.1)	7(0.14)	7(0.08)	0.289
ICD Implanted (n)	4(0.03)	3(0.06)	1(0.01)	0.143*

Values are n (%). * indicates the Fisher's exact probability test was used.
AF, atrial fibrillation; ICD, implantable cardioverter-defibrillator; MACCE, major adverse cardiac and cerebrovascular events; VT, ventricular tachycardia; VF, ventricular fibrillation.

Table S6. Outcome and events of HCM patients according to sex

Clinical events	Total (n=135)	Male (n=107)	Female (n=28)	*P*-value
MACCE events (n)	50(0.37)	36(0.34)	14(0.5)	0.111
Cardio-death (n)	4(0.03)	4(0.04)	0(0)	0.580*
Progress heart failure (n)	19(0.14)	13(0.12)	6(0.21)	0.227*
VT or VF (n)	8(0.06)	7(0.07)	1(0.04)	0.553*
Stroke (n)	3(0.02)	2(0.02)	1(0.04)	0.505*
Syncope (n)	13(0.1)	8(0.07)	5(0.18)	0.143*
AF (n)	14(0.1)	9(0.08)	5(0.18)	0.166*
ICD Implanted (n)	4(0.03)	3(0.03)	1(0.04)	1*

Values are n (%). * indicates the Fisher's exact probability test was used.
AF, atrial fibrillation; ICD, implantable cardioverter-defibrillator; MACCE, major adverse cardiac and cerebrovascular events; VT, ventricular tachycardia; VF, ventricular fibrillation.

Table S7. Outcome and events of HCM patients according to LVEF of CMR.

Clinical events	Total (n=135)	LVEF≥50% (n=89)	LVEF<50% (n=46)	*P*-value
MACCE events (n)	50(0.37)	30(0.33)	20(0.44)	0.208
Cardio-death (n)	4(0.03)	2(0.02)	2(0.04)	0.600*
Progress heart failure (n)	19(0.14)	9(0.1)	10(0.22)	0.054
VT or VF (n)	8(0.06)	5(0.06)	3(0.07)	0.481*
Stroke (n)	3(0.02)	2(0.02)	1(0.02)	1.000*
Syncope (n)	13(0.1)	9(0.1)	4(0.09)	1.000*
AF (n)	14(0.1)	8(0.09)	6(0.13)	0.55*
ICD Implanted (n)	4(0.03)	3(0.03)	1(0.02)	1.000*

Values are n (%). * indicates the Fisher's exact probability test was used.

AF, atrial fibrillation; ICD, implantable cardioverter-defibrillator; MACCE, major adverse cardiac and cerebrovascular events; VT, ventricular tachycardia; VF, ventricular fibrillation.

Table S8. Characteristics of HCM patients according to combination of genotype and LGE.

	G+/L+ (n=27)	G+/L- (n=23)	G−/L+ (n=27)	G−/L− (n=58)	*P*-value
Demographics					
Sex, male (n)	19(0.7)	16(0.7)	23(0.85)	49(0.84)	0.246
Age (years, mean ± SD)	53.519 ± 12.442	50.87 ± 13.706	51.63 ± 10.87	52.914 ± 10.195	0.823
Age of first visit (years, mean ± SD)	50.37 ± 11.225	50.87 ± 13.706	50.926 ± 10.813	52.759 ± 10.178	0.766
BMI (kg/m^2, mean ± SD)	23.53(21.97,24.89)	23.53(21.01,24.09)	22.23(20.55,24.44)	24.14(22.21,25.2625)	**0.037$^{\#}$**
NYHA grade, III-IV(n)	8(0.3)	1(0.04)	12(0.44)	8(0.14)	**0.001***
Previous heart failure admission (n)	6(0.22)	0(0)	8(0.3)	6(0.1)	0.004*
Previous stroke (n)	0(0)	0(0)	0(0)	1(0.02)	0.637*
Previous VT or VF (n)	1(0.04)	0(0)	2(0.07)	2(0.03)	0.465*
Previous syncope (n)	2(0.07)	3(0.13)	2(0.07)	1(0.02)	0.231*
Previous AF (n)	4(0.15)	0(0)	5(0.19)	5(0.09)	0.061*
Previous ICD (n)	0(0)	0(0)	1(0.04)	0(0)	0.355*
Diabetes (n)	0(0)	0(0)	1(0.04)	1(0.02)	0.540*
Hypertension (n)	7(0.26)	2(0.09)	8(0.3)	3(0.05)	**0.006***
Baseline Echocardiogram					
MWT (mm, mean ± SD)	17.4 ± 4.898	17.057 ± 4.473	16.4 ± 4.39	18.534 ± 4.748	0.220
IVS (mm, mean ± SD)	16(14,17)	17(15,22)	16(14,19)	17(16,22.25)	0.313$^{\#}$
LA (mm, mean ± SD)	39.341 ± 6.63	37.109 ± 6.947	39.093 ± 6.488	38.995 ± 7.015	0.644
RV (mm, mean ± SD)	20.533 ± 3.555	19.643 ± 2.283	20.352 ± 3.621	20.488 ± 2.72	0.693
LVPW (mm, mean ± SD)	11.041 ± 2.006	11.217 ± 2.58	10.317 ± 1.841	11.305 ± 2.195	0.263
HOCM (n)	8(0.3)	4(0.17)	3(0.11)	14(0.24)	0.326*
RVH (n)	1(0.04)	0(0)	0(0)	0(0)	0.355*
SVH (n)	22(0.81)	21(0.91)	25(0.93)	52(0.9)	0.598*
ApHCM (n)	6(0.22)	2(0.09)	2(0.07)	6(0.1)	0.357*
MRI Baseline					

LVEF (%, mean ± SD)	55(41,62)	55(46,65)	55(28,62)	62(49.75,66)	**0.048#**
LVEDV (ml, mean ± SD)	133(110,190)	112(94,144)	138(126,240)	130.5(108.75,156)	**0.008#**
LVESV (ml, mean ± SD)	53(44,112)	45(39,82)	74(48,132)	48.5(39.75,64.25)	**0.017#**
LVM (g, mean ± SD)	171.674 ± 68.261	179.053 ± 59.475	175.741 ± 49.008	174.948 ± 66.968	0.982
CO (L/min, mean ± SD)	4.876 ± 1.040	4.913 ± 1.533	5.213 ± 1.751	5.319 ± 1.724	0.570
CI (L/min/m2, mean ± SD)	2.839 ± 0.477	2.853 ± 0.675	2.866 ± 0.916	3.016 ± 0.745	0.641
Baseline ECG					
AF (n)	3(0.11)	3(0.13)	5(0.19)	9(0.16)	0.879*
LBBB (n)	5(0.19)	0(0)	2(0.07)	1(0.02)	**0.015***
Abnormal T-wave inversion (n)	11(0.41)	13(0.57)	9(0.33)	31(0.53)	0.237
Pathological Q wave (n)	6(0.22)	3(0.13)	4(0.15)	10(0.17)	0.836
Laboratory Tests					
NT-proBNP (ng/ml, mean ± SD)	880(501.4,2799)	1117(360,4191)	1274(179,2711.24)	754(182.5,2711.24)	0.251#
cTnT (ng/ml, mean ± SD)	0.024(0.01,0.17)	0.027(0.001,0.16)	0.01(0,0.16)	0.04(0.01,0.495)	0.306#
Medicine					
Beta bloker	15(0.56)	19(0.83)	14(0.52)	45(0.78)	**0.019**
CCB	6(0.22)	2(0.09)	5(0.19)	3(0.05)	0.086*
ACEI/ARB	2(0.07)	1(0.04)	2(0.07)	1(0.02)	0.523*
ARNI	7(0.26)	0(0)	4(0.15)	2(0.03)	**0.002***
Diuretic	3(0.11)	0(0)	4(0.15)	2(0.03)	0.065*

# indicates the Kruskal-Wallis H test was used. * indicates the Fisher's exact probability test was used. AF, atrial fibrillation; ApHCM, apical ventricular hypertrophy cardiomyopathy; BMI, body mass index; CI, cardiac index; CK-MB, creatine kinase MB; CO, cardiac output; cTnT, cardiac troponin T; ECG, electrocardiogram; HOCM, hypertrophic obstructive cardiomyopathy; ICD, implantable cardioverter-defibrillator; IVS, interventricular septum thickness; LA, left atrial; LBBB, left bundle branch block; LGE, late gadolinium enhancement; LVEDV, left ventricular end-diastolic volume; LVEF, left ventricular ejection fraction; LVESV, left ventricular end-systolic volume; LVPW, left ventricular posterior wall thickness; LVM, left ventricular mass; MRI, magnetic resonance imaging; MWT, maximal wall thickness; NT-proBNP, N-terminal pro-brain natriuretic peptide; NYHA, New York Heart Association; RV, right ventricular; RVH, right ventricular hypertrophy SVH, septal ventricular hypertrophy; VT, ventricular tachycardia; VF, ventricular fibrillation.
